# The effects of the semirecumbent position on hemodynamic status in patients on invasive mechanical ventilation: prospective randomized multivariable analysis

**DOI:** 10.1186/cc12694

**Published:** 2013-04-26

**Authors:** Ivan Göcze, Felix Strenge, Florian Zeman, Marcus Creutzenberg, Bernhard M Graf, Hans J Schlitt, Thomas Bein

**Affiliations:** 1 Department of Surgery, University Medical Centre Regensburg, Franz-Josef-Strauss-Allee 11, D-93053 Regensburg, Germany; 2 University of Regensburg, Universitätstrasse 31, D-93053 Regensburg, Germany; 3 Centre for Clinical Studies, University Medical Centre Regensburg, Franz-Josef-Strauss-Allee 11, D-93053 Regensburg, Germany; 4 Department of Anaesthesiology, University Medical Centre Regensburg, Franz-Josef-Strauss-Allee 11, D-93053 Regensburg, Germany

## Abstract

**Introduction:**

Adopting the 45° semirecumbent position in mechanically ventilated critically ill patients is recommended, as it has been shown to reduce the incidence of ventilator-associated pneumonia. Although the benefits to the respiratory system are clear, it is not known whether elevating the head of the bed results in hemodynamic instability. We examined the effect of head of bed elevation (HBE) on hemodynamic status and investigated the factors that influence mean arterial pressure (MAP) and central venous oxygen saturation (ScvO2) when patients were positioned at 0°, 30°, and 45°.

**Methods:**

Two hundred hemodynamically stable adults on invasive mechanical ventilation admitted to a multidisciplinary surgical intensive care unit were recruited. Patients' characteristics included catecholamine and sedative doses, the original angle of head of bed elevation (HBE), the level of positive end expiratory pressure (PEEP), duration and mode of mechanical ventilation. A sequence of HBE positions (0°, 30°, and 45°) was adopted in random order, and MAP and ScvO2 were measured at each position. Patients acted as their own controls. The influence of degree of HBE and of the covariables on MAP and ScvO2 was analyzed by using liner mixed models. Additionally, uni- and multivariable logistic regression models were used to indentify risk factors for hypotension during HBE, defined as MAP <65 mmHg.

**Results:**

Changing HBE from supine to 45° caused significant reductions in MAP (from 83.8 mmHg to 71.1 mmHg, *P *< 0.001) and ScvO2 (76.1% to 74.3%, *P *< 0.001). Multivariable modeling revealed that mode and duration of mechanical ventilation, the norepinephrine dose, and HBE had statistically significant influences. Pressure-controlled ventilation was the most influential risk factor for hypotension when HBE was 45° (odds ratio (OR) 2.33, 95% confidence interval (CI), 1.23 to 4.76, *P *= 0.017).

**Conclusions:**

HBE to the 45° position is associated with significant decreases in MAP and ScvO2 in mechanically ventilated patients. Pressure-controlled ventilation, higher simplified acute physiology (SAPS II) score, sedation, high catecholamine, and PEEP requirements were identified as independent risk factors for hypotension after backrest elevation. Patients at risk may need positioning at 20° to 30° to overcome the negative effects of HBE, especially in the early phase of intensive care unit admission.

## Introduction

The semirecumbent position is an upright positioning of the head and torso at an angle of 45°. The effects of adopting the semirecumbent position in critically ill patients have been extensively investigated as a potential means of preventing ventilator-associated pneumonia (VAP). VAP develops in 5% to 25% of ventilated patients and it is associated with prolonged duration of mechanical ventilation, hospital stay, and increased morbidity and mortality [1-3].

Reflux of gastric contents and subsequent microaspiration of bacterial contaminated oropharyngeal fluids play crucial role in development of VAP [4]. Use of histamine-2 receptor blockers or proton pump inhibitors (PPI) increases gastric pH and enhances colonization with pathogens. The combination of a nasogastric feeding tube and the supine position facilitates gastroesophageal reflux and increases the volume of oropharyngeal fluids significantly. The incidence of VAP is independently associated with a supine (0°) head of bed position during the first 24 h of mechanical ventilation [5]. Nursing patients in the semirecumbent position substantially decreases the aspiration of gastric contents, and a randomized trial has confirmed that this significantly reduces the incidence of VAP [6-8].

Despite being widely adopted, there is still some uncertainty about the routine use of the upright position. Control groups in trials investigating head of bed elevation (HBE) were nursed supine at 0°, which does not reflect current practice. It is also not known whether elevating the head of the bed to 45° may cause hemodynamic instability [9].

We examined the influence of HBE on hemodynamic status in patients on invasive mechanical ventilation. After randomization to one of six possible sequences of positioning we assessed hemodynamic parameters and central venous oxygen saturation in each position and also examined the variables that might be independent predictors of hemodynamic changes.

## Methods

### Patients

Two hundred patients were recruited in the multidisciplinary surgical intensive care unit (ICU) of a tertiary care university hospital. The study was approved by the local Institutional Review Board (Ethikkommision Universität Regensburg, no 10-101-0280). The written consent of unresponsive patients was obtained either from them after they regained responsiveness or from their next of kin. All hemodynamically stable, mechanically ventilated patients over the age of 18 years with a central venous catheter situated in the superior vena cava on the ICU were eligible for inclusion in the study. Hemodynamic stability was defined as a stable mean arterial pressure by constant inotropic support without additional fluid administration. Patients with acute cardiovascular instability, or those with pump-driven circulatory or respiratory support, were excluded from the study. Also excluded were all patients in whom the supine position is contraindicated (for example, patients with traumatic brain injury), or those who were immobilized due to spinal injuries or unstable pelvic fractures.

The amount of positive inotropic support, length of mechanical ventilation, ventilation mode, or length of ICU stay did not influence inclusion or exclusion from the study. We chose 15 disease categories to describe the admission diagnoses. Severity of the illness was recorded using the Simplified Acute Physiology Score (SAPS II), which expresses the probability of mortality based on 12 variables including a combination of physiological, laboratory, and clinical data [10]. Hemodynamic parameters and central venous oxygen saturation were recorded in each position.

### Randomization

Sequences of six possible combinations of three positions (0°, 30°, and 45°) were determined: 0°, 30°, then 45°; 0°, 45°, then 30°; 30°, 0°, then 45°; 30°, 45°, then 0°; 45°, 0°, then 30°; 45°, 30°, then 0°. Each combination was randomly assigned a number from 1 to 200. An independent institution created the random order (randomlist) and randomization was made in blocks, to guarantee uniform distribution of positioning sequences. Patients included in the study received a number in order (from 1 to 200) that determined the sequence of positioning from the list.

### Intervention

To measure the degree of positioning we used the mini digital protractor BevelBox (Anyi Instrument Co. Ltd., China). The angle sensor was calibrated prior to each measurement.

In the assessment period before intervention, we recorded the following parameters in all patients: positioning degree (°); age (years); gender; weight (kg); height (m); admission diagnosis; SAPS II; duration of mechanical ventilation (h) for the time between admission to ICU and intervention; tidal volume (mL); positive end-expiratory pressure (PEEP) (cmH2O); ventilation mode (either pressure-controlled ventilation (PCV) or pressure support ventilation (PSV)); peak airway pressure (P_max_) (cmH2O); level of inotropic support; dose of propofol and sufentanil for sedation; fluid balance in the last 24 h (mL/24 h); and serum albumin (g/L), hemoglobin (g/dL), and C-reactive protein (CRP) (mg/dL) concentrations. Only patients who mantained the hemodynamic stability during the assessment period were eligible for the protocol administration. The first backrest position was then adopted according to the randomization sequence. After 3 min to allow for hemodynamic adaptation, heart rate, systolic and diastolic blood pressure, and mean arterial pressure were recorded. The blood pressure was measured in all patients via arterial catheter placed either in the radial or femoral artery. The correct position of an arterial pressure transducer was evaluated after each positioning maneuvre. Simultaneously, 2 mL of blood were taken via jugular or subclavian central line for assessment of central venous oxygen saturation. The central venous oxygen saturation (ScvO2) values were automatically transferred from the blood gas analyzer (Radiometer, ABL 800, Flex, Copenhagen, Denmark) via network cable to the digital patient record system (Metavision Suite, iMD-soft, Tel Aviv, Israel). Then, these parameters were recorded in exactly the same way for the second and third positions. After the study was complete, the patient was returned to their original position. While the study protocol was being enacted, no changes were made to the doses of vasopressor or sedative drugs or the ventilator settings, nor were additional fluids administered.

### Statistical methods

Continuous variables are presented as means and standard deviations (SD) or as median values and interquartile ranges (IQR: q3-q1); categorical variables are presented as absolute numbers and proportions. Normality was verified according to statistical parameters (mean, median, skewness, and kurtosis) and visually by Q-Q Plots. To analyze the impact of HBE (0°, 30°, and 45°) and of the covariables (as listed in Table 1) on the mean arterial pressure (MAP) and on the ScvO2, linear mixed models were used. According to our primary aim, we analyzed the differences in MAP and ScvO2 between the degrees of HBE without regard for additional covariates. We provided mean values and SD as parameter estimates and adjusted the post-hoc pairwise comparisons by the Tukey-Kramer method. In the further analysis, we calculated different bivariable models, each containing HBE, an additional covariable, and its interaction term. Afterwards a multivariable model was calculated including all variables with a *P *value < 0.1 according to the bivariable model. In all mixed models the Kenward-Roger approximation was used, while HBE was used as a repeated effect and the correlation structure between the degrees of HBE was specified as unstructured. The normality of the residuals was tested by means of Q-Q Plots. For graphical illustrations, box plots and scatter plots were used. Both univariable and multivariable logistic regression models were conducted to analyze the risk for a mean arterial pressure <65 mmHg (MAP <65 mmHg) and a central venous oxygen saturation <70% (ScvO2 <70%), respectively, in the 45° position, according to the measured variables. The multivariable models were built using backward selection according to the likelihood ratio. For all significant covariables, we calculated odds ratios (OR) and corresponding 95% confidence intervals (95% CI). All reported *P *values are two-sided, and a *P *value of 0.05 is considered the threshold of statistical significance. Since the assessment of the covariables was of purely explorative nature, no adjustment for multiple testing was done. Data entry and calculations were made with the software package SPSS 19.0 (IBM, Chicago, IL, USA), and the linear mixed model analyses were undertaken using the SAS 9.2 procedure PROC MIXED (SAS Institute, Cary, NC, USA).

**Table 1:** Baseline characteristics of study patients (*n *= 200).

	*n *(%)
**Admission diagnosis**	
Postoperative cancer surgery	52 (26.0)
Trauma (excluding TBI)	29 (14.5)
Cardiovascular/cardiogenic shock	29 (14.5)
Infection/sepsis	27 (13.5)
Acute respiratory failure	10 (5.0)
Chronic respiratory failure	2 (1.0)
Transplantation	20 (10.0)
CPR (reanimation)	1 (0.5)
Gastrointestinal	11 (5.5)
Liver failure	5 (2.5)
Intoxication	1 (0.5)
Shock/hemorrhage	4 (2.0)
Neurological/stroke	5 (2.5)
Others	4 (2.0)
**Sex**	
Male	131 (65.5)
Female	69 (34.5)
**Ventilator mode**	
Spontaneous PSV	74 (37)
Controlled PCV	126 (63)
	**Mean (SD)**
**Age **(years)	60.0 (15.9)
**BMI **(kg/m^2^)	27.3 (5.9)
**SAPS II**	39.0 (11.8)
**Average backrest elevation **(°)	25.6 (6.3)
**Peak pressure **(cm H_2_O)	20.0 (4.6)
**Fluid balance last 24 h **(mL)	620 (1102)
**Albumin **(g/L)	22.5 (6.2)
**Hb **(g/dl)	9.5 (1.9)
	**Median (IQR)**
**Ventilator hours **(h)	24 (55)
**Tidal volume **V_t _(mL)	495 (141)
**PEEP **(cm H_2_O)	6 (4)
**Norepinephrine **(μg/kg/min)	0.07 (0.12)
**Propofol **(mg/kg/min)	0.02 (0.03)
**Sufentanil **(μg/kg/min)	0.01 (0.01)
**CRP **(mg/L)	92.9 (145.8)

BMI, body mass index; CPR, cardiopulmonary resuscitation; CRP, C-reactive protein; Hb, hemoglobin; Pmin, max, minimum and maximum; PCV, pressure-controlled ventilation; PEEP, positive end-expiratory pressure; PSV, pressure support ventilation; SAPS II, Simplified Acute Physiology Score; TBI, traumatic brain injury.

## Results

In total, 202 patients were recruited but on two occasions the study protocol was abandoned because of severe hypotension requiring volume and inotropic resuscitation. The baseline characteristics of all patients are shown in Table 1.

**Figure 1:**
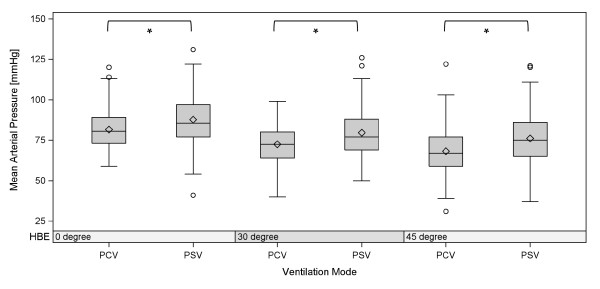
**Effect of PCV vs. PSV on mean arterial pressure with significant effect in all three degrees of backrest positioning, *P *< 0.001**. *P < 0.001: influence of ventilation mode on mean arterial pressure according to the linear mixed model. PCV: pressure controlled ventilation; PSV: pressure support ventilation.

**Figure 2:**
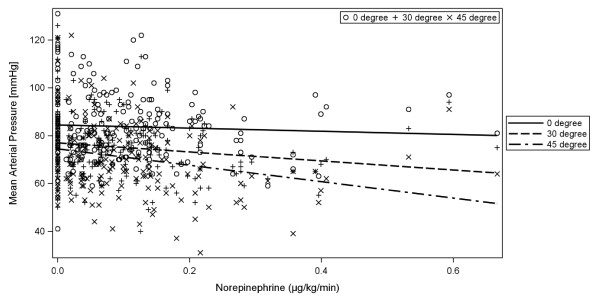
**Effect or norepinephrine on mean arterial pressure; *P *= 0.012**. Despite the high variability, there is an clear tendence to lower MAP by higher dose of norepinephrine. This effect is significantly stronger in the 45° position then in the full horizontal position; norepinephrine and its interaction with HBE, *P *= 0.005. HBE: head of bed elevation; MAP: mean arterial pressure.

There were significant differences between the three HBE positions for MAP (*P *< 0.001) as well as for ScvO2 (*P *< 0.001, Table 2). Only HBE from 0° to 30° was not associated with a significant decrease in ScvO2. For MAP as the dependent variable, in each of the bivariable models with the fixed factor HBE and an additional covariate, HBE stayed significant in all models and the covariates SAPS II, norepinephrine as well as its interaction with HBE (norepinephrine*HBE), ventilation mode, sufentanil, and propofol were found to be significant (Figures 1 and 2). In the subsequent multivariable model the variables HBE, norepinephrine*HBE, and ventilation mode had significant effects (Table 3).

**Table 2:** Influence of HBE on MAP and ScvO2.

	HBE (mean (SD))			*P *values^a^
	
	0°	30°	45°	Global (0° *vs*. 30°; 0° *vs*. 45°; 30° *vs*. 45°)
**MAP (mmHg)**	83.8 (14.5)	75.1 (13.1)	71.1 (15.2)	<0.001 (<0.001; <0.001; <0.001)
**ScvO2 (%)**	76.1 (8.0)	75.6 (8.2)	74.3 (9.0)	<0.001 (0.26; <0.001; 0.001)

HBE, head of bed elevation; MAP, mean arterial pressure; ScvO2, central venous oxygen saturation.

^a^According to Proc Mixed with Tukey-Kramer adjusted *P *values for pairwise comparisons.

**Table 3:** Variables influencing MAP.

	Bivariable model^a^ *P *value	Multivariable model^b^ *P *value
**HBE**	<0.001	<0.001

**SAPS II**	0.023	n.s.
**Norepinephrine (μg/kg/min)**	0.012	n.s.
Norepinephrine*HBE	0.005	0.005
**Ventilation mode**	<0.001	<0.001
**Sedation sufentanil (μg/kg/min)**	0.027	n.s.
**Sedation propofol (mg/kg/min)**	0.034	n.s.

^a^All bivariable models include HBE as a factor.

^b^The multivariable model contains all variables with a *P *value < 0.1.

HBE, head of bed elevation; n.s., not significant; SAPS II, Simplified Acute Physiology Score.

Taking ScvO2 as the dependent variable, HBE remained significant in all bivariable models and the covariates time of ventilation and its interaction with HBE and norepinephrine were also significant. In the multivariable model a significant effect was found for HBE, time of ventilation, time of ventilation and its interaction with HBE, norepinephrine, and ventilation mode (Table 4).

**Table 4:** Variables influencing ScvO2.

	Bivariable model^a ^*P *value	Multivariable model^b ^*P *value
**HBE**	<0.001	0.003

**Time of ventilation (h)**	n.s.	0.036
Time of ventilation*HBE	0.035	0.035
**Norepinephrine (μg/kg/min)**	0.006	0.009
**Ventilation mode**	n.s.	0.039

^a^All bivariable models include HBE as a factor.

^b^The multivariable model contains all variables with a *P *value < 0.1.

HBE, head of bed elevation; n.s., not significant; ScvO2, central venous oxygen saturation.

To calculate the risk of MAP falling below 65 mmHg in the 45° position, the group of patients who maintained MAP above this threshold was compared with those who did not (MAP <65 mmHg group). In the univariable comparison between both groups we found significant differences in: mean PEEP level, mean P_max_, mean dose of norepinephrine, mean initial backrest position, and ventilation mode (Table 5). In the univariable comparison of groups with ScvO2 <70% and ScvO2 >70% in the 45° position, none of the variables showed significant differences.

**Table 5:** Univariate logistic regressions on high-risk (MAP <65) *versus *low-risk (MAP >65) patients in the 45° position.

		MAP >65 mmHg (*n *= 128) Mean (SD)	MAP <65 mmHg (*n *= 72) Mean (SD)	OR	*P *value
**Age **(years)		59.8 (16.0)	60.5 (15.7)	1.00	0.77
**BMI**		27.1 (5.0)	27.7 (7.2)	1.02	0.52
**SAPS II**		37.9 (11.1)	40.9 (12.6)	1.02	0.09
**Measured backrest elevation **before intervention (°)		26.6 (6.2)	23.6 (6.0)	0.92	**0.002**
**Peak pressure **(cm H_2_O)		19.3 (4.6)	21.3 (4.4)	1.10	**0.005**
**Fluid balance last 24 h **(mL)		565 (1105)	718 (1097)	1.00	0.35
**Albumin **(g/L)		22.6 (6.3)	22.3 (6.2)	0.99	0.76
**Hb **(g/dL)		9.6 (2.0)	9.4 (1.8)	0.92	0.30
**CRP **(mg/L)		115.2 (92.1)	116.6 (97.3)	1.00	0.92
		**Median (IQR)**	**Median (IQR)**		
**Ventilation hours **(h)		24 (55)	28 (55)	1.00	0.45
**Tidal volume **(mL)		490 (147)	502.5 (140)	1.00	0.92
**PEEP **(cm H_2_O)		6 (3)	7.5 (5)	1.13	**0.013**
**Norepinephrine **(μg/kg/min)		0.06 (0.12)	0.09 (0.16)	1.04^a^	**0.005**
**Propofol **(mg/kg/min)		0.02 (0.03)	0.02 (0.02)	1.07^a^	0.44
**Sufentanil **(μg/kg/min)		0.01 (0.01)	0.01 (0.01)	1.00^b^	0.99
**Epinephrine **(μg/kg/min)		0.00 (0.00)	0.00 (0.00)	1.02^b^	0.33
**Dobutamin **(μg/kg/min)		0.00 (0.00)	0.00 (0.00)	1.28	0.37
**CRP **(mg/L)		103.5 (145.8)	88.4 (154.4)	1.00	0.92
		**Cases (%)**	**Cases (%)**		
**Ventilation mode**	Spontaneous (PSV)	57 (44.5%)	17 (23.6%)	1.61	**0.004**
	Controlled (PCV)	71 (55.5%)	55 (76.4%)		

^a^Per 0.01 unit change.

^b^Per 0.001 unit change.

BMI, body mass index; CRP, C-reactive protein; Hb, hemoglobin; IQR, interquartile range; MAP, mean arterial pressure; OR, odds ratio; PCV, pressure-controlled ventilation; PEEP, positive end-expiratory pressure; PSV, pressure support ventilation; SAPS II, Simplified Acute Physiology Score; SD, standard deviation.

In the multivariable logistic regression model, patients in the semirecumbent position had an OR for MAP <65 mmHg of 1.03 by raising the dose of norepinephrine (per 0.01 μg/kg/min increase, 95% CI 1.01 to 1.06, *P *= 0.023), of 1.13 by raising the level of PEEP (per 1 cmH_2_O increase, 95% CI 1.02 to 1.26, *P *= 0.0019) and of 2.33 if they were ventilated in pressure-controlled compared with pressure support mode (95% CI 1.16 to 4.69, *P *= 0.017). Moreover, patients with higher backrest elevation before the intervention were at a significantly lower risk of MAP <65 mmHg in the 45° position (OR 0.93, 95% CI 0.88 to 0.98, *P *= 0.005) (Table 6).

**Table 6:** Multivariate logistic regression model on high-risk patients (MAP <65) in the 45° position.

	OR (95% CI)^a^	*P *value
**Norepinephrine (μg/kg/min)**	1.03^b ^(1.01, 1.06)	0.023
**PEEP**	1.13 (1.02, 1.26)	0.019
**Backrest elevation (°)**	0.93 (0.88, 0.98)	0.005
**Ventilation mode^c^**	2.33 (1.16, 4.69)	0.017

^a^Odds ratio and 95% confidence interval.

^b^Per 0.01 unit change.

^c^Spontaneous (0), controlled (1).

PEEP, positive end-expiratory pressure.

## Discussion

This is the first prospective randomized self-controlled study evaluating the influence of upright positioning on hemodynamic stability. We found that increasing the angle of HBE to 30° and 45° is clearly associated with significant decreases in MAP when patients are mechanically ventilated. Moreover, the semirecumbent 45° position appears to cause significant falls in ScvO2. This suggests that the recommended semi-upright (30°) and upright (45°) positions may not be feasible in some mechanically ventilated patients.

Increasing the elevation of the head of the bed induces a gravitational transfer of blood from the upper body and central circulatory compartment towards the abdomen and lower limbs. This pooling of blood in the extremities reduces systemic venous return to the right heart and reduces cardiac output. Upright positioning may be responsible for lower mean circulatory pressure in ventilated patients, even if current evidence is lacking. Giving additional fluid boluses to increase circulating volume or increasing vasopressor support can reverse all these hemodynamic changes [11-14].

In our study upright body positioning at 45° was associated with sustained drop of MAP <65 mmHg in 72 (36%) patients. In addition, ScvO2 fell to below 70% in 62 (31%) patients. A MAP >65 mmHg and ScvO2 >70% are widely accepted and recommended hemodynamic parameters for adequate tissue perfusion and oxygenation in critically ill patients, targets that were not achieved by approximately one-third of our patients in the 45° position [15].

Our results showed significantly higher values for MAP and ScvO2 in the supine position. This may reflect the short-term effect of an improved circulation profile with higher systemic venous return, higher cardiac output, and, as a consequence, improved oxygen delivery. However, the supine position is clearly associated with increased incidence of ventilator-associated pneumonia, lung de-recruitment, and hypoxemia [16,17]. Any short-term positive effect on MAP and ScvO2 in the supine position is likely to be outweighed by respiratory complications in the longer term.

Our results suggest that critically ill patients in early phase after admission who require PCV and inotropic support are at high risk of a significant decrease in ScvO2 by increasing backrest position. Patients who required long-term ventilation showed significantly higher ScvO2 levels at the time of the study. They may have been in the recovery phase, with positive cumulative fluid balance and more cardiovascular reserve, lower oxygen demand, and less invasive mechanical ventilation than patients in the acute phase.

The dose of norepinephrine, level of PEEP, the PCV mode, and the angle of backrest elevation before the head of the bed was elevated can help identify patients at risk of hypotension (MAP <65 mmHg) when they are moved into the 45° position. The hemodynamic effect of PEEP on MAP is well known. Increased PEEP levels raise intrathoracic pressure, decrease right and left ventricular afterload and contractility leading to lower systemic blood pressure, especially in hypovolemic patients [18-20]. We found that raising the level of PEEP by 1 cmH_2_O creates a relative risk of hypotension (MAP <65 mmHg) of 1.13 in the 45° position, but also that increasing norepinephrine dose by 0.01 μg/kg/min is associated with a relative hypotension risk of 1.03. Increasing dose of norepinephrine reduces unstressed volume and vascular responsiveness. The greatest risk was PCV, which was associated with a relative risk of 2.33 for MAP <65 mmHg in the semirecumbent position. There are several reasons why the spontaneous (PSV) ventilator mode may be less deleterious on MAP during backrest elevation. Spontaneous inspiration with diaphragmatic contraction decreases intrathoracic pressure and hence systemic venous return to the heart is increased [21]. Furthermore, there is clear evidence that spontaneous breathing improves oxygenation and is associated with better systemic, hepatic, and intestinal blood flows [22,23].

Van Nieuwenhoven and colleagues have evaluated the feasibility of adopting the semirecumbent position in the daily routine for ventilated critically ill patients. They found that the targeted upright position of 45° was not attained for 85% of the study time in the semirecumbent group. Average elevations in the study group were 28.1° and 22.6° at days 1 and 7, respectively [24]. We measured the level of backrest elevation before starting intervention. Our findings support the previous published data with mean average backrest elevation of 25.6°. Moreover, the results showed that patients who developed a MAP <65 mmHg in 45° position had been nursed at significantly lower angles of backrest elevation before the intervention when compared with those whose MAP was maintained above 65 mmHg (23.6° *versus *26.6°). This suggests that patients at high risk of hypotension in the semirecumbent position are those who are routinely nursed below the recommended 30° to 45° of backrest elevation.

According to current published data, raising the head of the bed to 30° and 45° significantly increases the peak interface pressure between the skin at the sacral area and support surfaces in healthy volunteers, and in this context decubitus ulcers still remain a concern [25]. Positioning patients in the semirecumbent position is also associated with significant changes in intra-abdominal pressure [26-28]. We did not measure these variables in the present study. Other potential limitations are that our study was conducted in one center, a surgical ICU with a higher proportion of postoperative patients undergoing relatively short durations of PCV ventilation before the study. Our results may therefore be more generalizable for patients in the early acute phase after admission to the ICU rather than long-term ventilated ICU patients. Furthermore, due to the relatively low proportion of patients with acute respiratory failure, the strong effect of pressure controlled ventilation could have been caused by vast intrathoracic transmission of airway pressure. Therefore our results may have potential limitation if applied to patients with significantly reduced lung compliance. Finally, we only evaluated the hemodynamic response to HBE over 3 min. We found that most hemodynamic changes occurred within 30 s to 90 s, not dissimilar to the time taken for cardiac stroke volume to change after passive leg raising [29,30]. We believe that within the constraints of our protocol we detected all acute hemodynamic changes associated with altering the angle of the backrest, but cannot determine whether these changes would be maintained over time and what the longer-term consequences of these changes are.

Based on current evidence, the semirecumbent position clearly prevents VAP when compared to full horizontal position. Nevertheless, it is still unclear whether the 45° degree backrest elevation as originally reported by the Drakulovic's study is feasible, and whether lower inclination of head of bed could still be beneficial. The future studies may systematically address safety of the HBE and identify the safest, most effective head of bed orientation for patients on invasive mechanical ventilation.

## Conclusions

Adopting the 45° semirecumbent position is strongly associated with decreases in MAP and ScvO2 in mechanically ventilated patients. Patients who are sedated, undergoing pressure-controlled ventilation, with higher SAPS II scores, receiving elevated levels of PEEP or higher dose of norepinephrine are at greatest risk of hypotension. They may need positioning at 20° to 30° to overcome the negative influences of backrest elevation on hemodynamic stability, especially in the early phase of critical illness.

## Key messages

• Elevating the head of the bed to 45° is associated with significant decreases in MAP and ScvO2 in mechanically ventilated patients.

• Pressure-controlled ventilation, increasing SAPS II score, sedation, high catecholamine, and PEEP requirements are independent risk factors for hypotension after backrest elevation.

• Patients at risk may need positioning at 20° to 30° to overcome the negative effects of HBE, especially in the early phase of ICU admission.

## Abbreviations

ARDS: acute respiratory distress syndrome; BMI: body mass index; CI: confidence interval; CPR: cardiopulmonary resuscitation; CRP: C-reactive protein; HBE: head of bed elevation; ICU: intensive care unit; MAP: mean arterial pressure; OR: odds ratio; PCV: pressure controlled ventilation; PEEP: positive end expiratory pressure; PSV: pressure support ventilation, SAPS II: simplified acute physiology score; ScvO2: central venous oxygen saturation; VAP: ventilator-associated pneumonia.

## Competing interests

The authors declare that they have no competing interests.

## Authors' contributions

TB was responsible for the study design. FS and IG were responsible for administering the protocol. FZ, FS, IG, and TB performed the data analysis and were responsible for interpretation of data. IG, FZ, and TB drafted the manuscript. MC, BMG, and HJS critically reviewed the manuscript. All authors have given final approval of this version of the manuscript.
